# Herpetic uveitis caused by herpes simplex virus after cataract surgery in a patient without prior viral keratitis or uveitis: a case report

**DOI:** 10.1186/s12886-022-02326-w

**Published:** 2022-03-05

**Authors:** Feng Hu, Wenxue Guan, Yongpeng Zhang, Xiaoyan Peng

**Affiliations:** 1 Beijing Tongren Eye Center, Beijing Tongren Hospital, Capital Medical University, Beijing, China; 2 Beijing Institute of Ophthalmology, Beijing, 100005 China; 3 Beijing Ophthalmology and Visual Science Key Laboratory, Beijing, China; 4 Beijing Ophthalmology and Visual Science Key Laboratory, Beijing Institute of Ophthalmology, Beijing Tongren Eye Center, Beijing Tongren Hospital, Capital Medical University, 17 Hougou Lane, Chongnei Street, Beijing, 100005 P. R. China

**Keywords:** Herpetic uveitis, Anterior uveitis, Intraocular surgery, Ocular hypertension, Phacoemulsification surgery, Herpes simplex virus

## Abstract

**Background:**

To report a case of herpetic uveitis caused by herpes simplex virus after cataract surgery in a patient without prior viral keratitis or uveitis.

**Case presentation:**

A 70-year-old female was referred to our clinic with a 16-day history of acute blurry vision with painful redness in the right eye. She accepted cataract surgery for the right eye ten days before initial of ocular symptoms. There was significant inflammation in anterior chamber of the right eye. Retina exam showed moderate dense vitreous opacity but not necrotic or focal retinal lesion in the right eye. The aqueous humor collected from the right eye was positive for herpes simplex virus (HSV) DNA by PCR. The diagnosis of herpetic uveitis in the right eye was made due to clinical presentations and aqueous humor examination.

**Conclusion:**

Herpetic virus reactivation might occasionally occur after intraocular surgery in patients without prior ocular viral diseases, inducing atypical postoperative intraocular inflammation.

## Background

Postoperative intraocular inflammation is a leading cause of human discomfort, delayed recovery, and reduced visual acuity (VA) in patients after cataract surgery [1]. Postoperative intraocular inflammation is caused by non-infectious and/or infectious factors. Non-infectious intraocular inflammation usually presents as cells and/or flare in the anterior chamber and is relived with local steroid and/or non-steroid therapy. Infectious endophthalmitis after cataract surgery is caused by an invasion of bacteria/fungus during the operation [2]. Recurrence of herpes zoster disease after phacoemulsification surgery has been reported as common in eyes with herpes zoster related keratitis and/or uveitis (40.4%); this is especially common in those with a shorter period of quiescence and a greater number of recurrences before surgery [3]. Herein, we report a case of herpetic uveitis caused by herpes simplex virus (HSV) occurring 10 days after cataract surgery in a patient without a prior history of viral keratitis or uveitis. In this HSV uveitis case, the presence of significant fibrinous inflammation in the anterior chamber and vitreous body was unusual and delusory, partially resembling endophthalmitis. We assumed that HSV activation might have aggravated the postoperative inflammation after cataract surgery, inducing the combined clinical picture of HSV anterior uveitis and endophthalmitis.

## Case presentation

A 70-year-old woman was referred to our clinic for blurry vision with painful redness in the right eye for 1 month. She had undergone phacoemulsification and intraocular lens implantation in the right eye 10 days before the onset of ocular symptoms. On day 1 post-operation, her best corrected visual acuity (BCVA) in the right eye was 20/20. Then, on day 10 post-operation, she complained of acute blurry vision and painful redness; her intraocular pressure (IOP) was 35 mmHg in the right eye, and there was mixed conjunctival congestion and corneal edema. However, no keratic precipitates, flare, or cells in the anterior chamber were observed. She received focal prednisolone acetate and brimonidine eyedrops for the right eye. On day 17 post-operation, her VA in the right eye was 20/33, and the IOP in the right eye was 11 mmHg, and she underwent retro-hemispheric injection of 20 mg of triamcinolone acetonide. On day 26 post-operation, her VA in the right eye was reduced to 20/333, and the IOP was 7 mmHg, vitreous opacity was subsequently recorded in her medical chart. On day 27 post-operation, the patient underwent an intravitreal vancomycin injection in the right eye. On day 34 post-operation, fibrinous exudation in the anterior chamber in the right eye was recorded. She was referred to our clinic as her VA was still declining. Her past medical and family history were unremarkable, except for hypertension for 3 years. She had no history of prior herpetic keratitis or anterior uveitis.

**Fig. 1:**
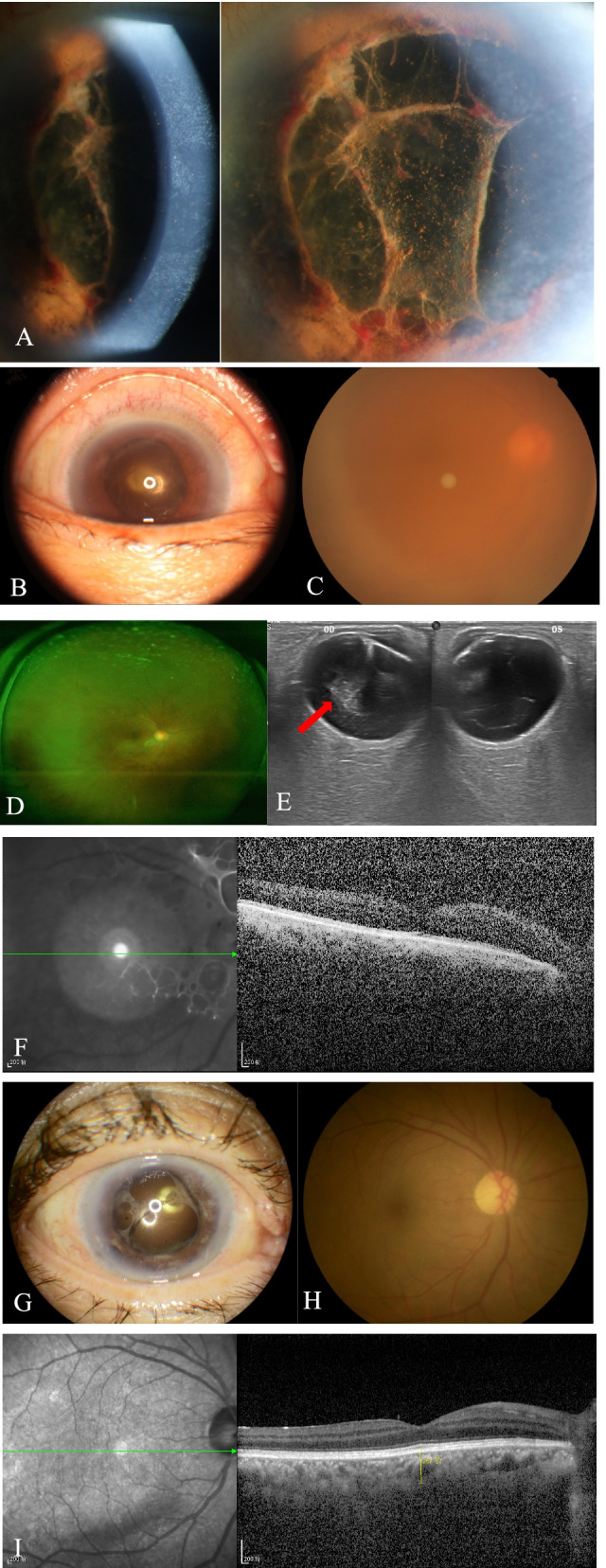
Imaging of slit lamp photograph, external ocular, fundus, wide-field fundus, OCT and ocular ultrasound of a 70-year-old patient with herpetic uveitis. **A** Slit lamp photograph revealed corneal epithelial erosion, corneal edema, pigmented keratic precipitates (KP), pigmented fibrin attached on the anterior surface of intraocular lens, hemorrhage of iris, and posterior synechia. **B** External imaging showed corneal edema. **C** Fundus photograph of the right eye was obscure due to vitreous opacity. **D** Wide-field fundus photograph showed no necrotic or focal retinal lesion in the right eye. **E** Ocular ultrasound showed dense vitreous opacity in the right eye (red arrow). **F** OCT scan of the right eye reveled vitreous opacity. **G** Three months after the antiviral therapy, corneal edema and vitreous opacity vanished. **H** Fundus photograph of the right eye demonstrated the vanishment of vitreous opacity three months after the antiviral therapy. **I** OCT scan of the right eye showed normal structure in the macular area

Upon initial examination, her visual acuity was 20/400 OD and 20/20 OS. The IOP was 8 mmHg in the right eye and 15 mmHg in the left eye. An examination of the anterior segment of the right eye revealed mixed conjunctival congestion, corneal edema, pigmented keratic precipitates, 1 + cells in the anterior chamber, posterior synechia, pigmented fibrin attached on the anterior surface of the intraocular lens, and hemorrhage of the iris (Fig. 1A and B). A retinal exam showed dense vitreous opacity (Fig. 1C) but no necrotic or focal retinal lesion in the right eye (Fig. 1D). Results of the anterior segment and retina exam of the left eye were unremarkable. Ocular ultrasound showed dense vitreous opacity in the right eye (Fig. 1E). OCT of the right eye showed that macular area was attached (Fig. 1F). The corneal thickness was 527 μm in the left eye and 444 μm in the right eye. The corneal endothelial cell density was 1,584/mm^2^ in the left eye, and not detected in the right eye.

The bacterium and fungal culture results for the vitreous biopsy collected from the right eye were negative. The aqueous humor collected from the right eye was positive for HSV by next-generation sequencing analysis, and no other virus, bacteria or fungus was detected. HSV-1 was further confirmed by a real-time-polymerase chain reaction (PCR) test of aqueous humor collected from the right eye with a titer of 9.42 × 10^4^/ mL. A diagnosis of herpetic uveitis in the right eye was made based on the clinical presentation and diagnostic tests results of the intraocular fluid. The patient underwent intravitreal ganciclovir (2 mg/0.1 mL) injections once weekly for 4 weeks and received oral valaciclovir (0.5 g 3 times daily) for two months. Three months after starting the antiviral therapy, her VA in the right eye had improved to 20/33, and the inflammation in the anterior chamber and vitreous body had vanished (Fig. 1G-I).

## Discussion

We report a case of herpetic uveitis caused by HSV that occurred shortly after phacoemulsification cataract surgery in an immunocompetent older female patient without a prior history of viral keratitis or uveitis.

Herpes zoster ophthalmicus (HZO) is caused by the reactivation of the varicella zoster virus and affects the ophthalmic division of the trigeminal nerve. Ocular involvement has been reported in 30%-78% of HZO patients, with common manifestations including conjunctivitis, keratitis, uveitis, and trabeculitis [4]. A recurrence of kerato-uveitis after cataract surgery has been reported to be common in patients with a prior history of HZO. The duration between cataract surgery and the recurrence of HZO is usually long. Lu et al. reported that the recurrence of HZO occurred mostly within the first 2 years after cataract surgery [3], while, according to another retrospective cases series, the recurrence of HZO could occur as long as 5 years after cataract surgery [5]. In the present case, the diagnosis of herpetic uveitis was confirmed by both next-generation sequencing analysis and PCR test of the aqueous humor, and the patient had a good response to antiviral therapy. New information provided by this case report was that virus activation or reactivation should be considered a potential cause of intraocular inflammation after cataract surgery.

The inducement for reactivation of the latent herpetic virus remains unclear. Multiple local effects including ultraviolet light [6], excimer laser [7], timolol [8], latanoprost [9], epinephrine [10], and corticosteroids [11] have been reported as risk factors for the recurrence of herpetic keratitis. Yttrium aluminum garnet laser peripheral iridotomy has similarly been reported as a risk factor for the reactivation of herpetic anterior uveitis. Research shows that pro-inflammatory cytokines are released following laser iridotomy and may play a role in the pathogenesis of recurrent herpetic uveitis [12]. It is well known that phacoemulsification cataract surgery could trigger non-infectious ocular inflammation due to unavoidable tissue damage [13]. A report by Al-Ani also demonstrated a greater recurrence of HSV ocular diseases in the first year following cataract surgery in patients with prior HSV-related keratitis and/or anterior uveitis [14]. Aggravated postoperative inflammation secondary to the activation of the herpetic virus might be associated with an atypical presentation of the presence of significant fibrinous inflammation in the anterior chamber.

Herpetic anterior uveitis is characterized by iris atrophy, acutely elevated IOP, scattered keratic precipitates, a dilated distorted pupil, and corneal involvement [15]. Elevated IOP is the most common ocular complication in patients with herpetic anterior uveitis (75%), followed by keratitis (59%), posterior synechia (34%), cataract (32%), and glaucoma (15%) [16]. Although it is reported that patients with HSV anterior uveitis often show significant inflammation in the anterior chamber [17], persistent high IOP with minimal anterior uveitis has also been documented as a less common presentation of herpes simplex uveitis after cataract surgery [18]. The present case was atypical, given the presence of significant fibrinous inflammation in the anterior chamber.

Acute-onset postoperative endophthalmitis is defined as endophthalmitis occurring ≤ 30 days after cataract surgery [19]. The mean duration between cataract surgery and the diagnosis of endophthalmitis in such cases is 8 days [20]. Risk factors for endophthalmitis after cataract surgery include advanced age, immunocompromised status, the presence of a septic focus in and around the eye, a posterior capsular break, and wound leakage [21]. Postoperative endophthalmitis is characterized by significant inflammation in the anterior chamber and dense vitreous opacity. In recent years, molecular diagnostic techniques for the intraocular fluid have been used for the auxiliary diagnosis of endophthalmitis and viral uveitis, which have high sensitivity and specificity [22]. Diagnostic techniques involve methods such as PCR and next-generation sequencing. Pathogen-directed PCR has been a powerful tool for the diagnosis of intraocular infections. For PCR, primer pairs specific for selected organisms are used to amplify DNA or RNA from those organisms. However, a primary limitation of pathogen-directed PCR is that only organisms preselected for screening can be detected. Next-generation sequencing is a newer method for evaluating infections, with which one can sequence DNA fragments from a biopsy of infected intraocular fluid [23].

In the present case, a diagnosis of attenuated endophthalmitis should be considered. However, it seemed unlikely that a final diagnosis of attenuated endophthalmitis due to bacterial and/or fungal infection was correct because of the negative results for both vitreous sample culture and the next-generation sequencing test of the aqueous humor; as well as the good prognosis attained without antibiotics or antifungal therapy. Postoperation non-infectious inflammation due to surgery attacks is common in patients after cataract surgery. In this case, the activation of HSV may aggravate postoperative inflammation in the anterior chamber and vitreous body, which resembling the clinical picture of endophthalmitis.

## Conclusions

We found that herpetic virus reactivation might occasionally occur after intraocular surgery in patients without prior ocular viral diseases, inducing atypical postoperative intraocular inflammation.

## Data Availability

All data supporting our findings are contained within the manuscript.

## Abbreviations

HSV: Herpes simplex virus
BCVA: Best corrected visual acuity
IOL: Intraocular lens
IOP: Intraocular pressure
KP: Keratic precipitates
VA: Visual acuity
HZO: Herpes zoster ophthalmicus
VZV: Varicella zoster virus
AU: Anterior uveitis
YAG: Yttrium aluminum garnet
